# Are the health messages in schoolbooks based on scientific evidence? A descriptive study

**DOI:** 10.1186/1471-2458-11-54

**Published:** 2011-01-26

**Authors:** Inés M Barrio-Cantalejo, Luisa M Ayudarte-Larios, Mariano Hernán-García, Pablo Simón-Lorda, José Francisco García-Gutiérrez, Jesús Martínez-Tapias

**Affiliations:** 1 Continuum Training and Research Department. Hospital of Baza, Spain; 2 Nursing Supervisor in Virgen de las Nieves Hospital, Granada, Spain; 3 Lecturer in Andalusian Public Health School, Granada, Spain; 4 Medical director in Santa Ana Hospital. Motril, Granada, Spain

## Abstract

**Background:**

Most textbooks contains messages relating to health. This profuse information requires analysis with regards to the quality of such information. The objective was to identify the scientific evidence on which the health messages in textbooks are based.

**Methods:**

The degree of evidence on which such messages are based was identified and the messages were subsequently classified into three categories: Messages with high, medium or low levels of evidence; Messages with an unknown level of evidence; and Messages with no known evidence.

**Results:**

844 messages were studied. Of this total, 61% were classified as messages with an unknown level of evidence. Less than 15% fell into the category where the level of evidence was known and less than 6% were classified as possessing high levels of evidence. More than 70% of the messages relating to "Balanced Diets and Malnutrition", "Food Hygiene", "Tobacco", "Sexual behaviour and AIDS" and "Rest and ergonomics" are based on an unknown level of evidence. "Oral health" registered the highest percentage of messages based on a high level of evidence (37.5%), followed by "Pregnancy and newly born infants" (35%). Of the total, 24.6% are not based on any known evidence. Two of the messages appeared to contravene known evidence.

**Conclusion:**

Many of the messages included in school textbooks are not based on scientific evidence. Standards must be established to facilitate the production of texts that include messages that are based on the best available evidence and which can improve children's health more effectively.

## Introduction

### Quality of health education in the School

Health education forms a part of the compulsory education curriculum in Spain, either as explicit content within a given area of knowledge or as supplementary content in any area or didactic act [1]. In order to encourage development in this area, with regards to both explicit and supplementary health education, various didactic methodologies have been promoted, which are more unidirectional in the first instance, and more participative and interactive in the second instance [2]. This two-fold approach to health education, particularly in the case of supplementary education, has given rise to a situation wherein practically all textbooks contain messages relating to health, irrespective of their subject matter. The profuse levels of information relating to health in school books require rigorous analysis with regards to the quality and suitability of such information [3]. Moreover, such analysis is indispensable as many of the health messages are formulated as advice or recommendations addressing the children directly. Certain studies have evaluated the quality of this information, measuring the effectiveness of messages aimed at illness prevention within the school [4] or the fostering of healthy habits [5]. Extracurricular programs that have been set in motion in schools by third parties have also been evaluated [6]. On other occasions, studies have focused on identifying the health areas addressed in the books or the didactic methodology employed to expound such content. However, we have found a single study that examined the contents about sexuality of the textbooks used in Spanish secondary education analysing the scientific evidence that support them [7]. Furthermore, in view of the section of the population that is targeted by these messages and their importance in improving health awareness and healthy behaviour amongst children, we must ensure that these messages are based on the best available evidence derived from reliable scientific studies.

### Scientific evidence in the health messages in textbooks

Evidence-based Medicine (EBM) is a process for the critical evaluation and incorporation of scientific discoveries into the decision-making process within the healthcare sector. This process can be applied to any type of health-related action: diagnosis, therapy or preventative measures [8]. From this perspective, health-related messages in schools should also be filtered via EBM. Determining the level of evidence of messages included in textbooks is of vital importance. The appropriateness of including messages that will be learned by children in textbooks should be determined by the level of evidence on which such messages are based.

The research questions for this study is if the health-related messages in textbooks are based on scientific evidence. The objective was to identify the scientific evidence used as a fundament for health-related messages in textbooks employed within compulsory education in Spain, namely, Primary and Secondary Education. The use of EBM methodology may prove extremely useful when selecting content to be included in school books. Textbooks should only contain the most reliable affirmations in relation to health, that is, statements that are solidly based on scientific evidence. The results of this study may aid the authors and publishers of school books to improve content. In addition, our results may also help teachers to place greater importance on the assimilation of messages that are more firmly based on scientific evidence. Finally, this study may facilitate the adoption of behaviours amongst children and the acquisition of knowledge by children that have more consistently demonstrated a capacity to improve health and prevent illness.

## Methods

This is an observational, descriptive and analytical study that was carried out in the city of Granada.

### Study material

Between the 1^st ^of March 2006 and the 1^st ^of June 2007, textbooks used in primary and secondary schools in a district of the province of Granada were identified. Books written in languages other than Spanish, "Teacher Guides" and books containing no health related messages were excluded. A representative sample of books was selected: a proportion with a precision of 5% was estimated along with a bilateral confidence interval of 95%.

Within the framework of the study, *health-related messages *refer to any statement within a school book that addresses any health-related subject in the form of a recommendation or advice. Messages relating to four areas were excluded: a) Aspects of Anatomy or Physiology that are beyond all scientific doubt were ignored (*the heart has four chambers*). b) Respect for the environment (*preventing damage to the ozone, water saving, recycling, etc*) as it is currently difficult to evaluate the scientific evidence and effects on health of recommendations in this area; c) Psychological and Social well-being relating to living in a community and mutual respect, as such considerations are not easily transformed into research questions and we were unable to find studies relating to evidence in this regard (*in order to live together without conflict, we must respect one another*); d) Messages relating to personal hygiene and food hygiene that emphasised norms of urbanity or courtesy (*don't let your hair fall into your food, don't clean your mouth with the sleeve of your shirt, etc*).

### Instrument

The texts selected were reviewed in order to identify any health-related messages. We drew up a table to classify messages, recording information relating to the school year and subject matter of the textbook containing each message. In addition, the focus of the message was defined in accordance with the classification of health priorities for children and adolescents drawn up by the health authorities [9].

### Procedure

Each health-related message in the school books was transformed into a research question. In order to transform the messages into interrogations, where possible, the Patient-Intervention-Comparison-Outcomes (PICO) scheme, drawn up by Richardson et al was employed. However, as these authors drew up their scheme for question construction in a clinical setting, which clearly does not apply to schools, the scheme was slightly modified for the present study. In the present study, the following aspects were considered in each question: (P) the individual or group targeted by the health-related message (boys, girls, adolescents, etc); (I) the area of health that the message focuses on, normally in the form of advice or recommendations (the consumption of fruit and vegetables, physical exercise, road safety, etc); (C) comparison with behaviour that is detrimental to health or with the failure to abide by the advice (the absence of fruit and vegetables in the diet, a sedentary lifestyle, failure to wear a helmet, etc); and (O) associated beneficial effects where the advice is followed. An example of transformation of the messages into interrogations is: *Tobacco smoke produces severe diseases of the respiratory system *(message) in: *Does tobacco smoke produce severe diseases of the respiratory system? *(question)

### Finding the best available evidence in relation to the questions

To this end we employed the *Trip Database (TD)*, one of the most important Internet resources for searches relating to evidence-based medicine. In accordance with TD recommendations, searches were carried out using search terms relating to question content in conjunction with Boolean searching, parenthesis and stemming.

From the results obtained via the TD, we only considered those documents containing information that was classified in terms of evidence, such as clinical practice guides and systematic reviews. The full texts of the documents were located and thoroughly reviewed, searching for a response to each research question. In each case, we identified the institution that published the document and the uniform resource locator (*url*) providing a link to the full text.

### Data analysis

Results relating to the level of evidence were classified into three categories: a) *Messages with a level of evidence*: messages wherein the research question is answered in at least one of the consulted documents that also report on the level of evidence. b) *Messages based evidence without assigned level*: messages wherein the research question is answered in at least one of the consulted documents, which nevertheless fail to report on the level of evidence. c) *Messages with no known evidence*: messages wherein the research question is not addressed in any of the consulted documents.

In the case of the *Messages with a level of evidence *group, the level of evidence was informed in each case. As different institutions employ a wide variety of systems to classify the degree of evidence, this study employed its own classification system, synthesising the most widely accepted classifications. This study classifies the level of evidence into three groups - high, medium and low - which homogenise and respect the original classification hierarchies that are most widely employed. Table 1 summarises the classification system used in this study.

**Table 1:** Scale employed in the study to measure the level of evidence

	HIGH	MEDIUM	LOW
Canadian Task Force on Preventive Health Care	A	B	C, D, E, I
U.S. Preventive Services Task Force	A	B	C, D, I
Centre for Evidence-Based Medicine Oxford	A	B	C
Scottish Intercollegiate Guidelines Network (SIGN)	A, B	C	D
U.K. National Institute of Clinical Excellence (NICE)	A, B	C	D, BPP, IP
U.S. Agency for Healthcare Research and Quality (AHRQ)	A	B	C

The statistically analysis used was only descriptive. Frequencies, percentages and mean were calculated.

## Results

### Textbooks and messages studied

237 textbooks used in primary and secondary schools were identified. 129 of them met the inclusion criteria. A representative sample of 80 books was randomised via simple aleatory selection. All the books were manually revised and 1135 health-related messages were identified. Subsequent to the elimination of messages that did not meet the criteria for inclusion, 844 were studied.

### Subject and School year of the health-messages

Table 2 displays the characteristics of all messages according to subject matter: 291 not eligible messages were found. Of these, 79 were about Anatomy and Physiology, 58 about Social and Psychological Well-being, 93 about Respect for the Environment and 61 about Norms of Urbanity or Courtesy. Balanced Diet and malnutrition was the most frequent subject of the messages (264, 31.3%), followed by Protection against environmental agents (96, 11.4%).

**Table 2:** Selection and randomisation of messages classified by subject.

	**Total Messages 1135**	
	**n *(%)***^1^	
**Not eligible Messages**		**Randomised Messages**
**291 *(25.6)***		**844 *(74.4)***
Anatomy/Physiology		Pregnancy/Newborns
**79 *(27.1*)**		**20 (2.4)**
Social and Psychological Well-being		Balanced Diet/Malnutrition
**58 *(1.9)***		**264 *(31.3)***
Respect for the environment		Obesity/Being Overweight
**93 *(32)***		**24 (2.8)**
Norms of Urbanity or Courtesy		Physical Exercise
**61 *(21)***		**54 *(6.4)***
		Respiratory Illnesses
		**19 *(2.3)***
		Food Hygiene
		**68 *(8.1)***
		Vaccines/Medical Treatment
		**24 *(2.8)***
		Traffic Accidents
		**30 *(3.6)***
		Accidents in the Home
		**12 *(1.4)***
		Protection against environmental agents
		**96 *(11.4)***
		Alcohol
		**7 *(0.8)***
		Drugs
		**11 *(1.3)***
		Smoking **27 *(3.2)***
		Sexual Behaviour/AIDS
		**19 *(2.3)***
		Oral Health
		**32 *(3.8)***
		Personal Hygiene
		**68 *(8.1)***
		Rest/Ergonomics
		**69 *(8.2)***

^1 ^Frequency and percentage.

As Table 3 illustrates, the average number of messages per book amounted to 10.6, whilst the highest concentrations were found in the 3^rd ^year of secondary school (37.1) and in the subject Biology and Geology (32.5). The subject Environmental Knowledge (13.5) and the fourth year of primary education (19.1) ranked second in terms of the number of messages. The 18 health-related messages found in the 2 Social Sciences text books were not included as, focusing almost exclusively on respect for the environment, they failed to meet the criteria for inclusion.

**Table 3:** Books, messages and messages per book according to school year and subject.

			Books Analysed	Health Messages	Health Messages **per Book**^**2**^
			**n *(%)***^**1**^		
**Year of School**	Primary	1	14 *(17.5)*	83 *(9.8)*	5.9
		2	12 *(2)*	59 *(7)*	4.9
		3	10 *(12.5)*	113 *(13.4)*	11.3
		4	7 *(8.7)*	134 *(15.9)*	19.1
		5	8 *(10)*	62 *(7.6)*	7.8
		6	8 *(10)*	97 *(11.5)*	12.1
	Secondary	1	8 *(10)*	29 *(3.4)*	3.6
		2	4 *(5)*	7 (*0.8*)	1.8
		3	7 (*8.7*)	260 (*30.8*)	37.1
		4	2 (*2.5*)	-	
**Subject Matter**	Globalised Method		12 *(15)*	79 *(9.4)*	6.6
	Environmental Knowledge		32 *(40)*	432 *(51.2)*	13.5
	Spanish Language and Literature		11 *(13.7)*	28 *(3.3)*	2.5
	Mathematics		6 *(7.5)*	8 *(0.9)*	1.3
	Natural Sciences		7 *(8.7)*	33 *(3.9)*	4.7
	Social Sciences Geography and History		2 *(2.5)*	-	-
	Technology		2 *(2.5)*	4 *(0.5)*	2
	Biology and Geology		8 *(10)*	260 *(30.8)*	32.5
**TOTAL**			**80**	**844**	**10.6**

^*1 *^Frequency and percentage. ^***2 ***^Average number of messages per book.

Table 3 also provides information on the number of books and classified messages according to academic year and subject matter. 59 books (73.7%) and 548 messages (64.9%) were of Primary School and 21 (26.3%) and 296 (35.1%) respectively of Secondary School. The mean of messages for book in Primary School was 9.3 and in Secondary School 15.

### Scientific evidence of the health-messages

Table 4 provides information on the frequency and percentage of classified messages according to subject matter and level of evidence. As the table shows, almost 61% of the messages are based on documentary evidence, although information on the levels of evidence is not provided. Less than 15% fell into the category where the level of evidence was known and less than 6% were classified as possessing high levels of evidence.

**Table 4:** Messages according to subject and level of evidence.

SUBJECT MATTER	Messages with a level of evidence			Messages based evidence without assigned level	Messages with no known evidence
	HIGH	MEDIUM	LOW		
	n *(%)*^1^				
Pregnancy Newborns	7 *(35)*	4 *(20)*	4 *(20)*	5 *(25)*	-
Balanced Diet Malnutrition	11 *(4.2)*	-	4 *(1.5)*	204 *(77.3)*	45 *(17.1)*
Obesity	-	1 *(4.2)*	2 *(8.3)*	15 *(62.5)*	6 *(25)*
Physical Exercise	-	-	10 *(18.5)*	34 *(63)*	10 *(18.5)*
Respiratory Illnesses	-	-	1 *(5.3)*	7 *(36.8)*	11 *(57.9)*
Food Hygiene	-	-	1 *(1.5)*	51 *(75)*	16 *(23.5)*
Vaccines Medical Treatment	6 *(25)*	1 *(4.2)*	-	10 *(41.7)*	7* *(29.2)*
Traffic Accidents	-	-	11 *(36.7)*	17 *(56.7)*	2 *(6.7)*
Accidents in the Home	2 (*16.7)*	-	*1 (8.3)*	4 *(33.3)*	5 *(41.7)*
Protection against environmental agents	1 *(1)*	1 *(1)*	7 *(7.3)*	36 *(37.5)*	51 *(53.1)*
Alcohol	-	-	2 *(28.7)*	3 *(42.9)*	2 *(28.6)*
Drugs	-	-	3 *(27.3)*	7 *(63.64)*	1 *(9.1)*
Smoking	1 *(3.7)*	-	3 *(11.1)*	19 *(70.4)*	4 *(14.8)*
Sexual Behaviour AIDS	-	-	-	14 *(73.7)*	*4 (26.3)*
Oral Health	12 (*37.5)*	3 *(9.4)*	0	4 *(12.5)*	13 *(40.6)*
Personal Hygiene	6 *(8.8)*	2 *(2.9)*	15 *(22)*	34 *(50)*	11 *(16.2)*
Rest Ergonomics	-	-	1 *(1.5)*	49 *(71)*	19* *(27.5)*
	46 *(5.5)*^*2*^	12 *(1.4)*^*2*^	65 *(7.7)*^*2*^	513 *(60.8)*^*2*^	208 *(24.6)*^*2*^

1 Frequency and percentage of messages according to subject matter and level of evidence.

2 Frequency and percentage of all messages according to level of evidence.

* Messages contravenes known evidence

"Balanced diets and malnutrition" is the subject matter that is most often addressed in messages and also gives rise to the highest frequency of messages based on a given source of evidence, although the level of evidence is not known. This subject, along with messages concerning "Food and Hygiene", "Tobacco", "Sexual behaviour and AIDS" and "Rest and ergonomics", is based on a source of evidence in more than 70% of cases, although, once again, the level of evidence is not specified.

"Oral health" registered the highest percentage of messages based on a high level of evidence (37.5%), followed by "Pregnancy and newly born infants" (35%).

Almost a quarter of the messages (24.6%) are not based on any known evidence. In the case of "Respiratory illnesses" and "Protection against environmental agents" more than half the messages are not based on any known evidence (57.9% and 53.1% respectively) and in the case of "Oral Health", the number of messages not based on any known evidence accounts for 40% of the total. In two instances, according to the documents consulted, the messages appeared to contravene known evidence.

Table 5 provides information on the documents - Clinical Practice Guides (98 in total) and Systematic Reviews (9 in total) - that were consulted in order to answer the research questions linked to health-related messages in school books. Table 6 provides examples of messages with different levels of evidence and identifies the two messages that contravene evidence according to the documentary sources that were consulted.

**Table 5:** Consulted Guides and Systematic Reviews with *url *to facilitate their location.

INSTITUTION	TITLE	URL
**Systematic Reviews**		
CL	Safety education of pedestrians for injury prevention	http://www2.cochrane.org/reviews/en/ab001531.html
CL	Phytomedicines (medicines derived from plants) for sickle cell disease	http://www.ncbi.nlm.nih.gov/pubmed/15266534
CL	A systematic review of the interventions to promote the wearing of hearing protection	http://www.biomedexperts.com/Abstract.bme/18317609/A_systematic_review_of_the_interventions_to_promote_the_wearing_of_hearing_protection
CL	Water for preventing urinary calculi	http://www2.cochrane.org/reviews/en/ab004292.html
CL	School-based programmes for preventing smoking	http://health-evidence.ca/articles/show/15727
CL	School-based prevention for illicit drugs' use	www2.cochrane.org/reviews/es/ab003020.html
CL	Feverfew for preventing migraine	www.cochrane.org/reviews/en/ab002286.html
CL	Herbal therapy for treating osteoarthritis	www2.cochrane.org/reviews/es/ab002947.html
CL	European guidelines for the management of chronic non-specific low back pain	http://www.kovacs.org/Imagenes/EuropeanGuidelinesCHRONIC.LBP.pdf
**Clinical Practice Guides**		
NHS-NICE	Antenatal care routine care for the healthy pregnant woman	http://www.nice.org.uk/CG006
HS-CKS	Insomnia	http://cks.library.nhs.uk/insomnia/view_whole_guidance
RACGP	Guidelines preventive activities general practice	http://www.racgp.org.au/guidelines/redbook
NHS-CKS	Dyspepsia: pregnanacy associated	http://www.cks.nhs.uk/access?catalog=login&returnurl=http%3a%2f%2fwww.cks.nhs.uk%2fdyspepsia_pregnancy_associated
NHS-NICE	Postanatale care	http://guidance.nice.org.uk/CG37/guidance/pdf/English
WHO	Care of umbilical cord	http://www.who.int/reproductivehealth/publications/maternal_perinatal_health/MSM_98_4/en/
CTFPHC	Interventions to promote breast-feeding: applying the evidence in clinical practice	http://www.cmaj.ca/cgi/reprint/170/6/976.pdf
PHAC	Canadian Immunization Guide 7th Edition	http://www.phac-aspc.gc.ca/publicat/cig-gci/index-eng.php
NHS-CKS	Immunizations-Childhood Vaccination Programme	http://cks.library.nhs.uk/immunizations_childhood/view_whole_guidance
RCN	Children's services: acute health care provision	http://www.rcn.org.uk/publications/pdf/ChildhoodVaccinationFactfile.pdf
HHS	Preventive services for children and adolescents	http://www.guideline.gov/summary/summary.aspx?doc_id=11700&nbr=006047&string=preventive
WHO	Sleep problems	http://www.mentalneurologicalprimarycare.org/index.asp
AAP	The Teen Driver	http://aappolicy.aappublications.org/cgi/reprint/pediatrics;118/6/2570.pdf
CL	Bicycle helmets: it's time to use them	www.bmj.com/content/321/7268/1035.1.full.pdf
NHS - SIGN	Prevention and management of dental decay in the pre-school child	http://www.sign.ac.uk/pdf/sign83.pdf
NHS - SIGN	Preventing Dental Caries in Children at High Caries Risk	http://www.sign.ac.uk/pdf/sign47.pdf
AAP	Oral Health Risk Assessment Timing and Establishment of the Dental Home	http://aappolicy.aappublications.org/cgi/reprint/pediatrics;111/5/1113.pdf
AAPD	Guideline on Periodicity of Examination. Preventive Dental Services	http://www.aapd.org/media/Policies_Guidelines/G_Periodicity.pdf
AAPD	Clinical guideline on infant oral health care	http://www.aapd.org/media/Policies_Guidelines/G_InfantOralHealthCare.pdf
AAPD	Clinical guideline on adolescent oral health care.	http://www.aapd.org/media/Policies_Guidelines/G_Adoleshealth.pdf
NHS-CKS	Pruritus vulvae	http://cks.library.nhs.uk/pruritus_vulvae/view_whole_topic_review
NHS-CKS	Lice	http://cks.library.nhs.uk/head_lice/view_whole_topic_review
ICSI	Acne Management. Third Edition	http://www.icsi.org/acne/acne_management_3.html
HHS	Eye	http://www.guideline.gov/summary/summary.aspx?doc_id=11018&nbr=5798&ss=6&xl=999
DOH	Management and control of eye conditions at primary level	http://www.doh.gov.za/docs/factsheets/guidelines/eye2.pdf
NHS-CKS	External otitis	http://www.prodigy.nhs.uk/otitis_externa/view_whole_guidance
HPA	Guidance on Infection Control In Schools and other hile Care Settings	http://www.hpa.org.uk/web/HPAwebFile/HPAweb_C/1194947358374
CCHMC	Urinary tract Infection	http://www.cincinnatichildrens.org/health/info/urinary/home/uti-prevention.htm
HHS	Abrasion and laceration wound care: pre-school through grade twelve	http://www.guideline.gov/summary/summary.aspx?doc_id=10196&nbr=5380&ss=6&xl=999
NHS-CKS	Common cold.	http://www.cks.library.nhs.uk/common_cold/view_whole_topic_review
HHS	Environmental management of pediatric asthma	http://www.guideline.gov/summary/summary.aspx?doc_id=10019&nbr=5333&ss=6&xl=999
NHS-CKS	Smoking cessation	http://www.cks.nhs.uk/access?catalog=login&returnurl=http%3a%2f%2fwww.cks.nhs.uk%2fsmoking_cessation
NHS-NICE	Feverish illness in children	http://guidance.nice.org.uk/CG47/NICEGuidance/pdf/English
AAP	Office-Based conseling for injury prevention	http://aappolicy.aappublications.org/cgi/reprint/pediatrics;94/4/566.pdf
NHS-CKS	Sprains and strains	http://www.cks.nhs.uk/access?catalog=login&returnurl=http%3a%2f%2fwww.cks.nhs.uk%2fsprains_and_strains
HHS	Counseling to prevent skin cancer: recommendations and rationale	http://www.uspreventiveservicestaskforce.org/3rduspstf/skcacoun/skcarr.htm
DOH	Guidelines for School Programs To Prevent Skin Cancer	http://www.cdc.gov/mmwr/preview/mmwrhtml/rr5104a1.htm
HHS	Pediatric eye and vision examination	http://www.aoa.org/documents/CPG-2.pdf
CPS	Impact of media use on children and youth	http://www.cps.ca/english/statements/CP/pp03-01.htm
NHS-NICE	Prevention of healthcare-associated infections primary and community care	http://www.sempsph.com/sempsph/attachments/185_InfControl-Prevention-Community-PrimaryCare.pdf
USPSTF	Screening for Visual Impairment in Children Younger than Age 5 Years	http://www.ahrq.gov/downloads/pub/prevent/pdfser/visualser.pdf
NHS-CKS	Chest infection	http://cks.library.nhs.uk/chest_infections_adult/view_whole_topic_review
HHS	Hearing assessment in infants and children	http://www.guideline.gov/summary/summary.aspx?doc_id=3614&nbr==9992840&ss=6&xl
HHS	Physical activity in the prevention. treatment and rehabilitation of diseases	http://www.guideline.gov/summary/summary.aspx?doc_id=10477&nbr=5500&ss=6&xl=999
NHS-DH	Green book	http://www.dh.gov.uk/en/Publichealth/Immunisation/Greenbook/index.htm
HHS	Health professional's guide rehabilitation of the patient with osteoporosis	http://www.springerlink.com/content/4tlqvrqla0xyyrav/
NHS-CKS	Ankylosing spondylitis	http://www.cks.nhs.uk/access?catalog=login&returnurl=http%3a%2f%2fwww.cks.nhs.uk%2fankylosing_spondylitis
RCGP	Sexually Transmitted Infections in Primary Care	http://www.bashh.org/documents/702/702.pdf
NHS-CKS	Contraception	http://cks.library.nhs.uk/contraception/view_whole_guidance
HHS	Tabacco use prevention and cessation for adults and mature adolescents	http://www.guideline.gov/summary/summary.aspx?doc_id=5454&nbr=3731&ss=6&xl=999
NHS-NICE	Chronic obstructive pulmonary disease	http://www.nice.org.uk/nicemedia/pdf/CG012_niceguideline.pdf
AHA	Diet and Lifestyle Recommendations. Revision 2006	http://circ.ahajournals.org/cgi/content/full/114/1/82
AAP	Tobacco. Alcohol. and Other Drugs: The Role of the Pediatrician	http://www.pediatrics.org/cgi/content/full/115/3/816
NHS-CKS	Smoking cessation	http://www.cks.nhs.uk/access?catalog=login&returnurl=http%3a%2f%2fwww.cks.nhs.uk%2fsmoking_cessation
HHS	Prevention of Ventricular Remodeling. Cardiac Dysfunction. and Heart Failure	http://www.guideline.gov/content.aspx?id=23899&search=prevention+of+ventricular+remodeling.+cardiac+dysfunction.+and+heart+failure
NHS-CKS	Alcohol-problem drinking	http://cks.library.nhs.uk/alcohol_problem_drinking
NHS - SIGN	The management of harmeful drinking and alcohol dependence in primary care	http://www.sign.ac.uk/guidelines/fulltext/74/index.html
NZGG	Food and Nutrition Guidelines for Healthy Adolescents	http://www.nzgg.org.nz/guidelines/0066/Nutrition_Guidelines.pdf
RCGP	Guidance for working with cocaine and crack	http://www.rcgp.org.uk/PDF/drug_cocaine.pdf
HHS	Dietary recommendations for children and adolescents	http://www.guideline.gov/summary/summary.aspx?doc_id=8215&nbr
HPA	Smallpox	http://www.hpa.org.uk/Topics/InfectiousDiseases/InfectionsAZ/SmallpoxDR/Guidance/
NHS-CKS	Insect bites and stings	http://www.cks.nhs.uk/access?catalog=login&returnurl=http%3a%2f%2fwww.cks.nhs.uk%2finsect_bites_and_stings
NHS-CKS	Anemia - Iron deficency	http://www.cks.nhs.uk/anaemia_iron_deficiency/evidence/references#
NZGG	Food and nutrition guidelines for healthy adolescent	http://www.nzgg.org.nz/guidelines/dsp_guideline_popup.cfm?guidelineCatID=24&guidelineID=66
NHS-CKS	Gastroenteritis	http://www.cks.nhs.uk/patient_information_leaflet/gastroenteritis
AHA	Diet and Lifestyle Recommendations Revision 2006	http://circ.ahajournals.org/cgi/content/full/114/1/82
NHS - SIGN	Management of obesity in children and young people	http://www.sign.ac.uk/pdf/sign115.pdf
NGCH	Prevention and screening of colorectal cancer	http://www.guideline.gov/content.aspx?id=12796&search=prevention+and+screening+of+colorectal+cancer
CAS	HIV Transmisión: Guidelines for assessing risk. 5^th ^Edition	http://www.cdnaids.ca/web/repguide.nsf/pages/cas-rep-0307
NASPGHAN	Overweight children and adolescents	http://www.naspghan.org/user-assets/Documents/pdf/PositionPapers/2005-06-13_OverweightChildrenAdolescents.pdf
VSSGBI	The provision of vascular service 2004	http://www.vascularsociety.org.uk/Docs/Provision%20of%20Vascular%20Services.pdf
NGCH	Identifying and preventing overweight in childhood. Clinical practice guideline	http://www.guideline.gov/content.aspx?id=9370
NHS-NICE	Secondary prevention in primary-secondary care patients myocardial infarction	http://www.nice.org.uk/nicemedia/pdf/CG48NICEGuidance.pdf
NHS-CKS	Obesity	http://www.cks.nhs.uk/obesity
MJA	Nutrition and physical activity for Australian children	http://www.mja.com.au/public/nutrition/question2.html
AAP	Optimizing Bone Health and Calcium Intakes of Infants. Children. and Adolescents	http://aappolicy.aappublications.org/cgi/content/full/pediatrics;117/2/578
SACN	Salt and health	http://www.sacn.gov.uk/reports_position_statements/reports/salt_and_health_report.html
ADA	Nutrition and Athletic Performance	http://www.eatright.org/About/Content.aspx?id=8365
BCHM	B12 Deficiency - Investigation & Management of Vitamin B12and Folate Deficiency	http://www.bcguidelines.ca/gpac/pdf/b12.pdf
NHS-CKS	Constipation	http://cks.library.nhs.uk/constipation/view_whole_guidance
B	Mediterranean diet evidence	http://www.medicine.ox.ac.uk/bandolier/band114/b114-2.html
AAP	Prevention of Rickets and Vitamin D Deficiency	http://aappolicy.aappublications.org/cgi/content/full/pediatrics;111/4/908
NHS-DH	Coronary Heart disease	http://www.dh.gov.uk/prod_consum_dh/groups/dh_digitalassets/@dh/@en/documents/digitalasset/dh_4057519.pdf
DOH	Guidelines for the management and health Surveillance of food handlers	http://www.doh.gov.za/docs/factsheets/foodhandlers.pdf
RCN	Malnutrition: What nurses working with children and young people	http://www.rcn.org.uk/__data/assets/pdf_file/0007/78694/003032.pdf
APsA	Practice guideline for the treatment of patients with eating disorders	http://www.ncbi.nlm.nih.gov/pubmed/10642782
NGCH	Prevention and management of obesity (mature adolescents and adults)	http://www.guideline.gov/content.aspx?id=14178&search=prevention+and+management+of+obesity+%28mature+adolescents+and+adults%29
NGCH	Increasing physical activity in schools: kindergarten through eighth grade	http://www.ncbi.nlm.nih.gov/pubmed/17536917
CDC	Guidelines for School and Community Programs to Promote Lifelong Physical Activity Among Young People	http://www.cdc.gov/mmwr/preview/mmwrhtml/00046823.htm
AAP	Activity Healthy living: prevention of childhood obesity through increased physical activity	http://www.aap.org/advocacy/releases/may06physicalactivity.htm
HPA	Guidelines for action in the event of a deliberate release	http://www.hpa.org.uk/infections/topics_az/deliberate_release/botulism/PDFs/botulism_guidelines.pdf%C2%A0
NHMRC	Staying Healthy in Child Care	www.nhmrc.gov.au/_files_nhmrc/file/publications/.../ch43.pdf
CPS	Bugs in our meal: Food for thought	http://www.cps.ca/english/statements/ID/ID01-02.htm
NGCH	Dietary guidelines for Americans. 2005	http://www.health.gov/dietaryguidelines/dga2005/default.htm
NHS-DH	Infection at work: Controlling the risks	http://www.dh.gov.uk/en/Publicationsandstatistics/Publications/PublicationsPolicyAndGuidance/DH_4070102

**Abbreviations**:

AAP: American Academy of Paediatrics

AAPD: American Academy of Pediatric Dentistry

ADA: American Dietetic Association

AHA: American Heart Association

APsA: American Psychiatrics Association

B: Bandolier

BCMH: Britisth Columbia-Ministry of Health

CAS: Canadian AIDS Society

CCHMC: Cincinnati Children's Hospital Medical Center

CDC: Centers of Disease Control and Prevention

CL: Cochrane Library

CPS: Canadian Paediatric Society

CTFPHC: Canadian Task Force on Preventive Health Care

DOH: Department of Health) of South Africa

HPA: Health Protection Agency

ICSI: Institute for Clinical Systems Improvement

NGCH: National Guidelines Clearinghouse

NHS-CKS: Clinical Knowledge Summaries. antes Prodigy

NHS-DH: Department of Health

NHS-NICE: National Institute for Health and Clinical Excellence

NHS-SIGN: Scotish Intercollegiate Guidelines Network

NHMRC: Australian National Health Medical Research Council

MJA: Medical Journal of Australia

NZGG: New Zeland Guidelines Group

PHAC: Public Health Agency of Canada

NASPGHAN: North American Society for Pediatric Gastroenterology. Hepatology. Nutrition

RCN: Royal Collage of Nursing

RACGP: The Royal Australian Collage of General Practitioner

RCGP: Royal College of General Practitioners

SACN: Scientific Advisory Committee of Nutrition

USPSTF: US Preventive Services Task Force

VSSGBI: The Vascular Surgical Society of Great Britain and Ireland

WHO: World Health Organization

**Table 6:** Examples of messages with different levels of evidence

LEVEL OF EVIDENCE	MESSAGE
HIGH	• Children should use toothpaste with fluoride
	• The Mediterranean diet helps to prevent excess cholesterol
	• Adopting habits such as not smoking prevents certain types of cancer
MEDIUM	• Sugar-free chewing gum helps to prevent tooth decay
	• After cleaning, cover a wound with a plaster or bandage
	• Pregnant women should visit the doctor once a month
LOW	• Moderate exercise strengthens the heart muscles and prevents illnesses affecting the circulatory system
	• Alcohol consumption leads to malnutrition and complaints affecting the alimentary canal
	• In Summer, apply sun cream to babies several times a day and after bathing
UNKNOWN	• Drinking two litres of water per day helps us to remain healthy
	• Children should always be assisted by an adult when cooking in order to avoid accidents in the home
	• Fruit should be thoroughly washed or pealed prior to consumption
NO EVIDENCE FOUND	• Do not swim after a meal. The digestive process may be affected, giving rise to a stomach cramp.
	• Breathing through the nose rather than the mouth prevents colds
	• An adequate intake of vitamin B lowers the probabilities of illness
EVIDENCE CONTRAVENED	• Wounds should be disinfected with peroxide
	• A period of inactivity should always be observed in the event of muscular injury

## Discussion

Attention should be drawn to the considerable number of health-related messages found in school books. This can be interpreted as a symptom of the high levels of observance of the official recommendation to include health education in all areas of the curriculum. However, a shadow is cast on this optimistic view when we consider the number of messages that are not based on scientific evidence. This raises questions in relation to the criteria that are employed to select messages for inclusion in school books. The fact that almost a quarter of the messages that were studied are not based on any documentary evidence (Guides and Systematic Reviews), suggests a certain degree of arbitrariness and a lack of clearly defined criteria to evaluate the importance of a health-related message. If we assume that the inclusion of a health-related message in the sources of evidence is indicative of their importance and suitability, our results show that far too many health-related messages aimed at school children are not firmly based on scientific evidence.

Nevertheless, the presence of health-related messages in guides and systematic reviews can not be used as the only means of justification for their inclusion in school books. This is due to the fact that clinical practice guides and systematic reviews entail a long preparation period that may significantly delay the publication and circulation of health information based on evidence. Moreover, the psychology of learning suggests that directing a health-related message towards children, even where the message is not based on sufficient scientific evidence, may aid the consolidation of healthy habits. For example, consulted documents recommend that children clean their teeth two times a day, a message that is based on a high level of evidence. However, the documents do not state that the cleaning process should be carried out after each meal. Nevertheless, recommending that teeth are cleaned immediately after each meal may help children to consolidate habits and routines that entail health benefits.

This paper does not intend to recommend the exclusion, at a general level, of messages that are not clearly based on evidence. However, we do feel that it is advisable to place less emphasis on messages that are not backed up by sources of evidence with a greater degree of frequency. For example, this is the case of messages relating to protection against environmental agents.

This study presents a number of shortcomings that bear mention. Considering clinical practice guides and systematic views as the only source of evidence may limit the scope of the study. Nevertheless, the tenets of care practice consider observance of the recommendations of a clinical practice guide or high quality systematic review to represent the most efficient method of ascertaining the best clinical course to follow [10].

Nor can we consider the exclusive use of the Trip Database as an evidence search engine as a shortcoming. Other search engines and other search strategies may produce different results. However, we decided to use this pre-filtered source of evidence and thereby ensure that all documents consulted were based on evidence that has been rigorously verified.

As conclusion, almost a quarter of messages included in school textbooks have an unknown scientific evidence. These messages are included in textbooks along with messages that are solidly based on scientific evidence. Children receive all of these messages and are unable to identify the importance or lack of importance of a given message or the extent to which it is based on evidence. The results of our study suggest that there is a need to establish standards that will enable the publishers who draw up the texts and the teachers who employ such texts to select and impart messages with solid scientific evidence.

Finally, this study has some practical implications: The results of our study demonstrate that there is a need to establish a mechanism that is capable of selecting messages on the basis of the degree to which they are based on scientific evidence. The publication of books that respect the criterion of a firm basing in scientific evidence with regards to the messages that they contain will facilitate the acquisition of knowledge amongst children that has consistently proved capable of improving health and preventing illnesses.

## Competing interests

The authors declare that they have no competing interests.

## Authors' contributions

IMB was the main researcher; she guided the study and wrote the first manuscript. MLA revised all textbooks and selected the health messages and reviewed the draft. MHG revised all text books and selected the health messages and reviewed the draft. PSL revised the sources of evidence (Guidelines and Systematic Review), analysed the evidence which support the health messages and reviewed the draft. JGG revised the sources of evidence (Guidelines and Systematic Review), analysed the evidence which support the health messages and reviewed the draft. JMT analysed the evidence which support the health messages and reviewed the draft. All authors read and approved the final manuscript.

## Pre-publication history

The pre-publication history for this paper can be accessed here:

http://www.biomedcentral.com/1471-2458/11/54/prepub
